# Visual adaptation and the amplitude spectra of radiological images

**DOI:** 10.1186/s41235-018-0089-4

**Published:** 2018-01-24

**Authors:** Elysse Kompaniez-Dunigan, Craig K. Abbey, John M. Boone, Michael A. Webster

**Affiliations:** 1 Department of Psychology, University of Nevada, Reno, NV USA; 2 Department of Psychological and Brain Sciences, University of California, Santa Barbara, CA USA; 3 Department of Radiology and Biomeidcal Engineering, University of California, Davis, CA USA

**Keywords:** Medical images, Adaptation, Spatial vision, Natural image statistics, Blur perception, Spatial contrast sensitivity, Visual search

## Abstract

We examined how visual sensitivity and perception are affected by adaptation to the characteristic amplitude spectra of X-ray mammography images. Because of the transmissive nature of X-ray photons, these images have relatively more low-frequency variability than natural images, a difference that is captured by a steeper slope of the amplitude spectrum (~ − 1.5) compared to the ~ 1/f (slope of − 1) spectra common to natural scenes. Radiologists inspecting these images are therefore exposed to a different balance of spectral components, and we measured how this exposure might alter spatial vision. Observers (who were not radiologists) were adapted to images of normal mammograms or the same images sharpened by filtering the amplitude spectra to shallower slopes. Prior adaptation to the original mammograms significantly biased judgments of image focus relative to the sharpened images, demonstrating that the images are sufficient to induce substantial after-effects. The adaptation also induced strong losses in threshold contrast sensitivity that were selective for lower spatial frequencies, though these losses were very similar to the threshold changes induced by the sharpened images. Visual search for targets (Gaussian blobs) added to the images was also not differentially affected by adaptation to the original or sharper images. These results complement our previous studies examining how observers adapt to the textural properties or phase spectra of mammograms. Like the phase spectrum, adaptation to the amplitude spectrum of mammograms alters spatial sensitivity and visual judgments about the images. However, unlike the phase spectrum, adaptation to the amplitude spectra did not confer a selective performance advantage relative to more natural spectra.

**Electronic supplementary material:**

The online version of this article (10.1186/s41235-018-0089-4) contains supplementary material, which is available to authorized users.

## Significance

Despite enormous advances in image processing and automation, interpretation of the majority of medical images still rests ultimately on visual judgments by radiologists. These judgments are challenging because the visual evidence for a diagnosis can be subtle and variable. Also, medical images have unique characteristics which are distinct from the diet of images we normally encounter in the natural visual environment. We hypothesize that differences in these characteristics are important, because they mean that vision is not inherently optimized for encoding or gaining information from medical images. Processes that might shape this encoding, by adjusting to the idiosyncratic properties of the images, are thus important for understanding both the limits and potential for perception and performance with medical images. We examine the role of visual adaptation in medical image perception. Adaptation routinely adjusts visual sensitivity in response to the stimuli we are currently exposed to. We ask how this adaptation adjusts to the characteristic amplitude spectra in full-field digital mammograms, a projection X-ray imaging modality, and in turn how these adjustments affect spatial sensitivity and the perception of blur. Our results are important for understanding the states of adaptation induced when observers inspect medical images and the impact of these states on how the images are perceived and interrogated.

## Background

The visual system continuously recalibrates to match the current stimulus environment (Webster, 2015). These adjustments are critical for optimizing visual coding for the widely varying properties of the scenes we encounter (Wainwright, 1999; Wark, Lundstrom, & Fairhall, 2007). Variations in the natural visual environment are known to provide a potent stimulus for adaptation (Simoncelli & Olshausen, 2001). Adjustments occur throughout the visual hierarchy and affect most if not all of the attributes we perceive, from the average light level to highly abstract properties of the visual world (Clifford et al., 2007; Kohn, 2007; Rieke & Rudd, 2009; Solomon & Kohn, 2014; Webster, 2011). Adaptation therefore plays a fundamental role in determining what we see and how well we can see it. In turn, understanding the states of adaptation induced by different stimulus contexts is critical for understanding the relevant operating states of the visual system – what and how we see within a given context – which may change dramatically from one situation to the next.

We examined how the visual system adapts within the context of medical image perception. Evaluations and diagnoses of radiological images still depend to a large extent on the visual judgments of radiologists and thus many studies have explored how these judgments are affected by known processes and constraints of human perception (Krupinski, 2011; Wolfe, Evans, Drew, Aizenman, & Josephs, 2016). Radiologists can spend hours at a time visually inspecting images. Thus, it is likely that they will adapt to the properties of these images and that this adaptation will change their sensitivity and perception. In previous work we explored the consequences of this adaptation by focusing on how observers (who were not trained radiologists) adapt to the structural properties of mammogram images (Kompaniez, Abbey, Boone, & Webster, 2013). Mammograms have characteristic variations in their textural properties corresponding to variations in tissue density. Radiologists classify breast texture in mammograms using the BI-RADS density scale which codes the tissue as one of four levels ranging from fatty (1) to dense (4) (American College of Radiology, 1998). In layman’s terms, the appearance of dense tissue can be thought of as being clouded with fibroglandular tissue. Fatty breasts contain little or none of these glandular “clouds,” although some clearly visible fibrous tissue may remain. Dense tissue is more likely to mask the presence of lesions and is associated with an increased risk for cancer, thus is more likely to lead to further diagnostic screening (Boyd, 2011; Boyd et al., 2007; Hersh & Marla, 2004). In our study, we found strong selective adaptation to these textural properties. Specifically, prior exposure to dense images caused an intermediate texture to appear more fatty and vice versa. Thus, adaptation could, in principle, have a significant effect on how mammograms are perceived and classified.

In a subsequent study, we also found that adaptation to the textures of mammograms impacted the ability to detect information in the images (Kompaniez-Dunigan, Abbey, Boone, & Webster, 2015). One postulated function of adaptation is to discount the expected properties of the visual environment, thus enhancing the visual salience of unexpected or novel information (McDermott, Malkoc, Mulligan, & Webster, 2010; Webster, 2014; Wissig, Patterson, & Kohn, 2013). This is essentially the problem confronting the radiologist, who must inspect medical images for the presence of aberrant or suspicious features. The properties of visual search in radiological settings has thus been a question of central interest (Bochud, Abbey, & Eckstein, 2004; Drew, Evans, Vo, Jacobson, & Wolfe, 2013; Eckstein, 2011; Horowitz, 2017; Jiang, Das, & Gifford, 2017; Krupinski, Berger, Dallas, & Roehrig, 2003; Kundel, Nodine, Conant, & Weinstein, 2007; Mello-Thoms, 2006; Nodine & Kundel, 1987; Wolfe et al., 2016). Adaptation could potentially facilitate performance by highlighting how an image differs from the image properties to which the observer is adapted. To test this, we added simulated lesions (Gaussian spots) at a random location in mammograms and then measured search times for detecting them. Search times were significantly faster when observers were first adapted to the images and this advantage was only found when adapting to the same class of images (dense or fatty) they were tested on, again pointing to selective adaptation to the textural properties of the images. Our results thus suggested that the textural characteristics of medical images provide a potent stimulus for adaptation, affecting both how the images are perceived and how efficiently they can be scanned for a feature like a lesion.

In the spatial frequency domain, the textural differences between the fatty and dense images primarily reflects differences in the phase spectra. Indeed, when we swapped the amplitude and phase spectra of fatty and dense images the after-effects followed the phase (Kompaniez-Dunigan et al., 2015). This was expected because the two classes of images have similar amplitude spectra. Importantly however, these amplitude spectra are highly unnatural (Burgess, Jacobson, & Judy, 2001). In the present study, our goal was to instead examine how observers adapt to this unnatural but characteristic property of medical images. Natural images tend to have an amplitude spectrum in which average contrast falls roughly inversely with spatial frequency or as 1/f, corresponding to a slope of − 1 on a log amplitude vs log frequency plot (Field, 1987; Tolhurst, Tadmor, & Chao, 1992; van der Schaaf & van Hateren, 1996). Individual images or image classes can vary in their specific slope, but the general trend of 1/f spectra is a ubiquitous characteristic of most natural scenes – in both space and time – and is a property that is thought to have shaped many aspects of spatiotemporal visual coding. For example, the spatial tuning of cells in both the retina and early visual cortex are well predicted from efficient coding of natural scene statistics, and suggest that vision is optimized for the natural visual environment (Atick, 1990; Field, 1987; Simoncelli & Olshausen, 2001).

In contrast, in medical images like mammograms, amplitude falls more rapidly with frequency, roughly with a slope of − 1.5 (Burgess et al., 2001). This steeper slope reflects more “blur” in the images, though it differs from the frequency filtering typical of optical blur, and thus presents the radiologist with stimuli that their vision should a priori be poorly matched for. However, blur itself is a salient attribute of images and a feature that can induce strong adaptation (Mon-Williams, Tresilian, Strang, Kochhar, & Wann, 1998; Pesudovs & Brennan, 1993; Sawides et al., 2010; Webster, Georgeson, & Webster, 2002). We therefore asked how exposure to the unnatural amplitude spectra or “blur” inherent in medical images affects the states of visual adaptation and the consequences of these states for visual judgments and performance limits with the images.

## Methods

### Participants

Observers included author EK and 14 undergraduate students who were unaware of the specific aims of the study. EK ran in all conditions while different students were tested in each of the individual experiments. All participants were “lay” observers with no training in radiology or medical image assessment and had normal or corrected-to-normal visual acuity.

### Apparatus

Stimuli were presented on a SONY 500 PS CRT monitor controlled by a VSG graphics card (Cambridge Research Systems). The monitor was calibrated and gamma-corrected based on measurements with a PR650 spectroradiometer (PhotoResearch).

### Stimuli

Mammogram images were obtained from an existing database of normal mammograms that included BI-RADS classifications provided previously by a radiologist (Chen, Abbey, Nosratieh, Lindfors, & Boone, 2012). Twenty images each were selected from the categories of fatty (BI-RADS score of 1) or dense (BI-RADS score of 4). For stimuli, we then selected random sections from within the mammogram. These sections were 256 × 256 pixels (experiments 1 and 2) or 600 × 800 pixels (experiment 3) and were chosen from random locations within the full 2560 × 3238-pixel image, with the provision that the chosen section fell entirely within a region of breast tissue. To remove differences in mean luminance and contrast, the 8-bit pixel values of the selected image patches were rescaled to have a constant average luminance of 37 cd/m^2^ on the display and a constant rms contrast of 0.38.

**Fig. 1:**
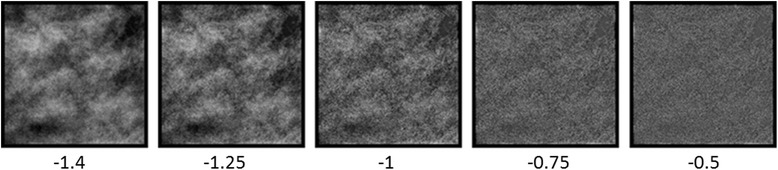
Example of an original image patch from a mammogram classified as dense, along with sharpened versions created by altering the slope of the average amplitude spectrum from the original slope (− 1.4) to a slope of − 0.5

To vary the amplitude spectra of the images, we processed the images in the frequency domain to alter the slope of the spectrum (Knill, Field, & Kersten, 1990; Tadmor & Tolhurst, 1994; Webster & Miyahara, 1997). Measurements of the best fitting slope to the original image patches showed that they had an average slope of α = − 1.4. To sharpen the spectra, we rescaled the original amplitude spectrum at each spatial frequency by an exponent of frequency so that the average slope had values of − 1.25, − 1, − 0.75, or − 0.5. This provided a set of adapting images that were either blurrier (original spectrum and − 1.25) or sharper (−0.75 and − 0.5) than the canonical 1/f spectrum (− 1) (Fig. 1). For test stimuli, we also generated arrays of images that varied the amplitude spectra of the images by varying the slope over a range of − 0.5 to − 1.5. The slope was varied in steps of 0.01 to create a finely graded series of 101 images spanning the range and centered on the 1/f slope. As before, the images were each rescaled to a constant mean and rms contrast after filtering.

### Procedure

Observers viewed the images binocularly in a darkened room and used a handheld keypad to record their responses. Adaptation to the amplitude spectra of the images was assessed with three different procedures.

**Fig. 2:**
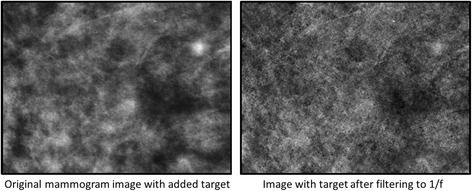
*Left*: original mammogram image section with a target (Gaussian blob) added in the *upper right quadrant*. The original image has a slope of − 1.5. *Right*: the image and target after filtering to a slope of − 1
  During a search trial, the static image was displayed on the screen with the onset signaled by a tone. The target location within the image was selected at random but was at least 0.55° from the vertical midline so that the side could be readily judged. Observers used a pair of buttons to respond as quickly as possible whether the target was on the left or right. A third button was also available for responding if the target could not be located. These instances were rare and the trials discarded, including the results for one of the fatty images for which correct detection was difficult. Observers searched for target in each of the 40 images (20 dense and 20 fatty) eight repeated times. To prevent learning, the images were shown rotated or reflected about the horizontal or vertical axis.
  Observers searched for the targets after adapting either to the original images or to the same set of images filtered to have a 1/f amplitude spectrum. Note that the filtering was done after adding the target and thus affected both the target and background, in order to simulate how the same image should appear under two different adaptation states (i.e. to the original or sharpened spectra) (Webster, 2014). There were four adapting conditions corresponding to the two image classifications (fatty or dense) and filtering (original or sharpened). Adaptation was initially for 300 s, during which a sequence of images from the set was shown with new images selected at random every 250 ms. Search trials were then presented interleaved with 4-s readaptation intervals. In each daily session, observers adapted to one condition (e.g. dense original) and then searched for the targets within the original image set or the sharpened image set (e.g. dense original or dense sharpened). Thus, the adaptation effects were compared only across the filtering to compare the effect of amplitude spectrum and not across the image type, which was the comparison assessed in our previous study (Kompaniez-Dunigan et al., 2015). To exclude the influence of unusually short or long reaction times, for each observer the response times for a given image and adapt condition were based on the median of their eight repeated settings. The reaction times were then averaged across the observers by taking the mean of the individual search times. Results shown are based on the average settings for seven observers.

1. *Perceived blur.* In the first case we measured how adaptation to the characteristic “blur” in medical images altered the perception of blur, by testing how adaptation to the original or filtered images biased the perceived amplitude spectra of the images. Judging when a complex texture like breast tissue in a mammogram appears in focus is a perceptually difficult task, since it requires comparing the image to some internal reference for subjective focus for an image that does not have distinct structure or features such as well-defined edges. To test for blur after-effects, we instead used a relative judgment in which observers adapted to both a blurred and sharpened image in two separate fields and then compared the relative appearance of a pair of test images shown within the same fields. The slopes of the test images were adjusted with a staircase until they appeared the same and the perceptual match was taken as the mean of the final six of ten reversals. This dual-adapt design had the advantage that the rms contrast between the two fields remained constant, so that perceptual differences could not be due to differences such as apparent contrast (Elliott, Georgeson and Webster, 2011). The design also provided a sensitive probe of any perceptual shift, since adaptation within each field should differentially bias the percepts, amplifying the perceptual difference between the test stimuli. To measure this difference, the levels of the two test images were yoked to vary symmetrically around a slope of − 1 (which corresponded to a match level or difference of 0 between the two test images). Participants judged whether the right image was more blurred or sharp relative to the left image. A “blurred” response caused the next displayed pair to be more sharpened on the right but more blurred on the left. (The stimulus directions corresponding to these responses could be learned quickly from whether the chosen response caused the two images to converge or diverge in appearance.)
  Observers viewed the display from a distance of 124 cm. The pair of images was shown in two 4° fields centered 2.2° to the left or right of the black fixation cross. The fields were also delimited by black borders from the 6.6° by 8.75° background, which had the same mean luminance and chromaticity as the images. During a run, observers first adapted for 60 s to either the gray fields (pre-adapt condition) or to adapting images shown within the fields (post-adapt). The adapt images filled the 4° fields (which were 228 × 228 pixels). However, their position was randomly jittered by ± 16 pixels every 100 ms in order to reduce the effects of local light adaptation. After the adapt phase, the test images were shown for 250 ms and interleaved with 4 s of readaptation, with 100-ms gray intervals between adapt and test images. This sequence repeated until the staircase completed. Observers made four repeated settings for each adapting condition, with separate settings measured for the dense image set and the fatty image set. The order of runs was counterbalanced across conditions.
2. *Contrast sensitivity.* To examine how adaptation influenced contrast thresholds, we measured detection thresholds for sinewave gratings after adapting to the mammogram images. For this experiment, the viewing distance was increased to 200 cm and the adapting images subtended 5° and were displayed in the center of the screen. Observers initially adapted for 120 s to an array of images drawn from one of three sets: the original dense mammogram images; the same images filtered to slope of − 1; or a gray field (used to measure threshold contrast sensitivity before adaptation). The fatty image set was not tested. A different adapting image from the dense set was cycled every 250 ms to avoid local light adaptation. After the initial adaptation period, a test grating was displayed for 500 ms, and alternated with 4-s readaptation intervals. The gratings were windowed by a fixed Gaussian envelope and displayed for 250 ms. 250-ms gray intervals separated each adapt and test stimulus. On each trial the orientation of the test grating was randomly set to 45° or 135° and participants indicated the orientation with a button press. The grating contrast was varied in 0.1 log unit steps with a three-down one-up staircase, with the run terminated after 11 reversals. Thresholds were calculated from the mean of the final eight reversals. Grating spatial frequency remained constant during a run and was in the range of 0.5–16 c/deg in one-octave steps across runs. Observers completed four repeated settings for each test frequency and adapting condition.
3. *Visual Search.* In the final condition, we examined whether prior adaptation to the amplitude spectra of the images could influence search times for detecting tumor-like targets presented within the images. For this experiment, the 600 × 800 sections from either fatty or dense mammogram images were shown on the full monitor screen from a distance of 260 cm, at which they subtended 6.6° by 8.75°. Targets were Gaussian spots (sd = 1.8°), which have been frequently used as targets in experiments examining detection in noise or medical images (Burgess, Li, & Abbey, 1997; Burgess, Wagner, Jennings, & Barlow, 1981). The spots were superimposed on the image by adding the luminance of the target to the image background (Fig. 2). The contrast of the targets was varied over a range of levels to vary the salience of the targets. These levels corresponded to peak increments in the linearized D/A levels above the background pixel level of 70, 90, 110, 130, or 150.

## Results

### Perceived blur

**Fig. 3:**
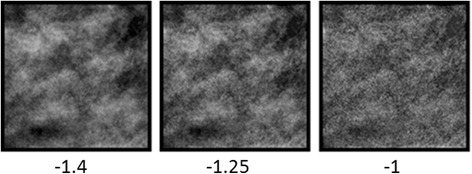
Still images illustrating blur after-effects in the images (the animated version of this figure is available to view in Additional file 1). The three adapting images with slopes of − 1.4, − 1.25, or − 1 are shown for 3 s, alternated with three test images all with the same slope of − 1.25 shown for 1 s. The after-effects are best experienced by continuously fixating the center image. The test images on the left and right should appear sharper and blurrier, respectively, relative to the central test image

Figure 3 provides an animation illustrating blur after-effects in the mammogram images. The three patches reproduce the three leftmost images from Fig. 1, showing a section from a dense mammogram with the original amplitude spectrum (slope = − 1.4) or sharpened to a slope of − 1.25 or − 1. These “adapt” stimuli are alternated in time with three identical “test” patches all with the middle slope of − 1.25. As the sequence is cycled, the physically identical test patches should appear perceptually different. Specifically, the left test patch alternated with the original mammogram image should appear sharper than the central patch, while the right patch alternated with the sharpened adaptor should appear blurrier. Thus, to match the appearance of the tests, observers would need to set the slope of the left image to be physically blurrier and the right image to be physically sharper.

**Fig. 4:**
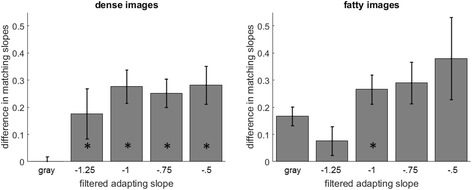
Blur after-effects for the mammogram images (original vs sharpened) displayed in the *left* and *right fields*. Settings are shown for the dense images (*left*) or fatty images (*right*). Each *bar* shows the physical difference between the slopes of the test images in sharpened vs original adaptor field required to match the perceived slope of the two images and correspond to the mean settings ± 1 standard error. The * symbol indicates adapting slopes where the matching slopes were significantly different from the baseline (no adapt) condition

Figure 4 shows measurements of the actual blur matches, by measuring how adaptation to the mammogram images biased the perceived spectral slope of a “focused” 1/f image. The values are based on the average settings for the five participants tested. Again, in this experiment a pair of test images were shown in the left and right fields and observers were required to adjust their relative slopes until they appeared the same. The slopes in the two images were yoked relative to − 1, so that an increase in one image’s slope was accompanied by a decrease in the other, and the two images were physically the same only when they both were 1/f. When adapted to the gray screen (pre-adapt baseline), the matches were close to − 1, showing that observers were sensitive to the relative slope differences in the images. However, after adapting to the original dense image (e.g. on the left) and a sharpened version of the image on the right, physically equal slopes appeared too blurred on the right and too sharp on the left. Thus, the perceived point of equality was biased toward sharpened settings on the right and vice versa on the left. The graphs plot the differences that needed to be introduced between the slopes of the test images in order to null the perceptual differences introduced by adaptation. After-effects were assessed with a two-way ANOVA (image slope by image type [dense or fatty]). This revealed a significant effect of slope (F(4,45) = 4.17, *p* = 0.006) while insignificant effects of image type (F(1,45) = 0.67, *p* = NS) and interaction (F(4,45) = 0.963, *p* = NS). Paired comparisons showed that for the dense images, settings were significantly biased in the expected direction compared to the neutral (gray field) adaptation and the differences were significant for the original vs all four sharpened adaptors (all *p* < 0.05; not corrected for multiple comparisons). The shifts were in the same direction for the fatty images, though in this case the differences reached significance only for the − 1 adaptor (t(4)-2.36, *p* = 0.038). As suggested by the pre-adapt settings, the weaker measured effects for the fatty images is possibly because observers were less reliable in making the relative blur judgments with these images. Nevertheless, the results suggest that observers were biased by adaptation to the blur in the mammogram images and that this adaptation altered their judgments of image focus.

### Contrast sensitivity

**Fig. 5:**
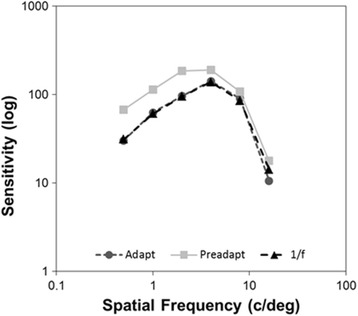
CSF following neutral, gray-field adaptation (*light gray squares*), adaptation to the original mammogram scans (*dark gray circles*), and adaptation to mammogram images filtered to have a slope of − 1 (*black triangles*). Points show the mean settings for the five observers

Figure 5 shows how adaptation to the mammogram images affected threshold contrast sensitivity. The illustrated values are again based on the average settings for the five observers tested, who each showed a similar pattern of after-effects on contrast sensitivity. Under neutral adaptation (to the gray field), the contrast sensitivity function (CSF) exhibits typical bandpass tuning with peak sensitivity for frequencies of ~ 2–4 c/deg (Kelly, 1975). In contrast, adaptation to the mammogram images produced marked and selective losses in sensitivity to low and medium spatial frequencies. As a result, the CSF more clearly peaks at ~ 4 c/deg and sensitivity to higher frequencies remained largely unaffected by the adaptation. This low frequency suppression suggests a more sharpened response to spatial frequency when observers are exposed to the mammograms and at first glance is in line with the changes in apparent suprathreshold blur revealed by the previous experiment. However, the changes in the CSF were indistinguishable when observers were instead adapted to the sharpened 1/f mammogram images. This is consistent with previous studies showing that adaptation to different amplitude spectra has a strong selective impact on lower spatial frequencies, but is not strongly selective for the specific spectral slope (Webster & Miyahara, 1997). This pattern was confirmed in a two-way ANOVA comparing frequency and adapting condition, which showed significant main effects of frequency (F(5,40) = 24.8, *p* < 0.001) and adapt (F(2,40) = 43.4, *p* < 0.01) as well as a significant interaction between the adapt condition and spatial frequency (F(10,40) = 2.10, *p* = 0.048). Pre-adapt thresholds differed from thresholds after adapting to both the original (F(1,29) = 68.9, *p* < 0.001) or sharpened images (F(1,29) = 73.5, *p* < 0.001), while the original and sharpened adapting conditions did not differ from each other (F(1,29) = 0.145, *p* = 0.71). Thus, the present results suggest that inspecting mammograms does alter the relevant CSF of the observer, but in ways that are similar to adaptation to images with natural amplitude spectra.

### Visual search

**Fig. 6:**
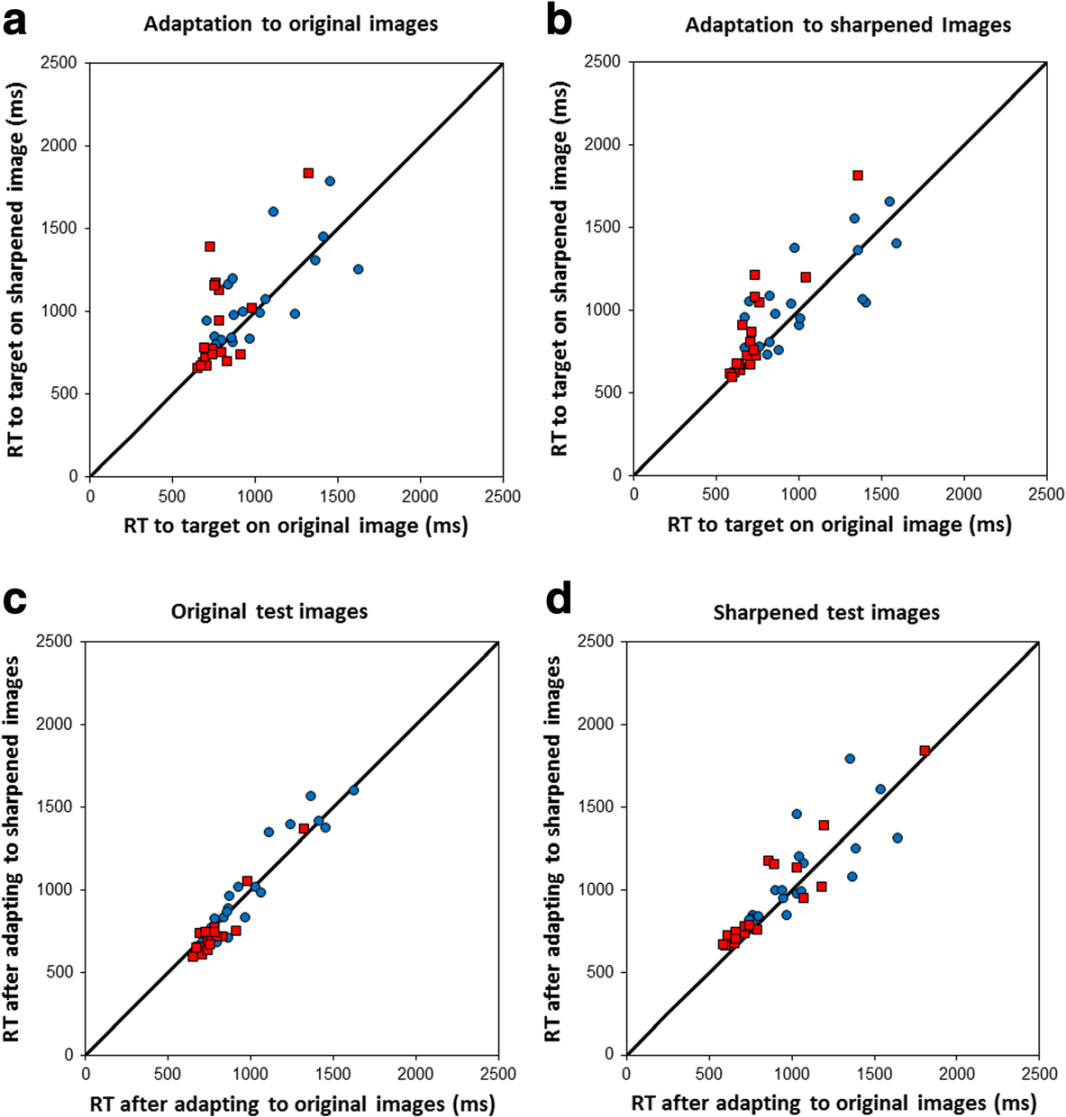
Average search times for detecting targets on the original or sharpened mammogram images, after adapting to the original images or sharpened images. *Points* plot the mean reaction times for individual fatty (*red squares*) or dense (*blue circles*) images. *Top*: comparison of search times on the sharpened vs original versions of the images after adapting to the original mammogram images (*left*) or sharpened images (*right*). *Bottom*: search times for adaptation on the sharpened images vs the original images, compared for the original test images (*left*) or sharpened test images (*right*)

The final experiment examined whether adaptation to the amplitude spectra of the images could influence the ability to detect targets in the images. However, in this case we did not observe clear effects of either the image filtering or the observer’s adapted state. Figure 6 shows mean reaction times for detecting the targets on the original or sharpened images. Specifically, the individual points show the search times for a given image and adapt condition based on the mean of the response times for the seven observers. Results are also shown separately for the fatty (red squares) and dense (blue circles) images. Average search times varied widely because of the variations in target contrast and overall was faster on the fatty images. This is consistent with the generally sparser structure in the fatty images, which makes it more likely that the targets, added at random locations, fell within an uncluttered region of the background, so that their local contrast was higher and thus reaction times shorter.

The top two panels of Fig. 6 compare the search times for sharpened vs original images when observers were adapted to the same background (original or sharpened). These panels thus highlight whether filtering the images (or adapting to the filtering) affects the speed of target detection. The changes with adaptation were assessed with a sign test comparing the number of times (images) for which search was faster for the original or sharpened image. For the dense backgrounds, sharpening the mammogram images (and targets) did not result in a significant change in the search times. Thus, these results suggest that the search performance was similar for the original and sharpened images and also that the relative sensitivity to the targets was not affected by the observer’s state of adaptation. For fatty backgrounds, search times instead became longer when the images were sharpened (RTs faster 26/38 times for the original images pooled across the two adapting conditions; *p* = 0.034). The scatter plots also suggest that this trend may be stronger for the fatty test images with the slowest search times, though we do not have sufficient data to formally assess this.

The lower two panels instead compare search times for the same images (either original or sharpened), across the two different adapting conditions (original or sharpened). These panels thus focus more closely on whether there is a selective effect of the adaptation. For the dense images there was again no significant change in the search times whether adaptation was to the same or different blur level that observers were searching on. For fatty images, we instead found a bias, but surprisingly in the wrong direction to the predicted effect. Specifically, the average search times were more often faster for the sharpened images after adapting to the original images (16/19 times, *p* = 0.004) or vice versa (15/19 times, *p* = 0.019). However, analyses of the settings for individual observers showed that this bias was significant only for three of the seven observers for the original test images and for only one of the seven for the sharpened images. Thus, whatever its basis, these effects were not robust and for both the dense and fatty images there was little evidence for a selective advantage of adapting to the backgrounds observers were searching on. These results thus differ from the adaptation effects we observed previously when adapting instead to the textural differences in the images, which again did show a selective advantage for finding the targets when observers were searching on the same (dense vs fatty) backgrounds they were adapted to (Kompaniez-Dunigan et al., 2015).

## Discussion

To summarize, our results reveal that adaptation to the characteristic amplitude spectra of mammogram images induces characteristic changes in suprathreshold blur perception and in threshold contrast sensitivity, but did not lead to improvements in target detection and salience. We consider each of these effects in turn and also consider how the different after-effects are related.

Blur after-effects of the kind we observed have now been widely studied and occur both when images themselves are blurred or when blur is introduced by the optical aberrations of the eye (Webster & Marcos, 2017). In fact, it is likely that this adaptation routinely functions to calibrate spatial vision and subjective image focus by discounting the retinal image blur introduced by the eye’s optics (Artal et al., 2004; Radhakrishnan, Dorronsoro, Sawides, Webster, & Marcos, 2015; Sawides, de Gracia, Dorronsoro, Webster, & Marcos, 2011). In most situations, it is the characteristics of the observer that are the primary sources of blur – i.e. their optical errors. Again, this is because the world typically has constrained spatial statistics such that the amplitude spectra of most scenes fall as roughly 1/f (Field & Brady, 1997; Tolhurst et al., 1992; van der Schaaf & van Hateren, 1996), and because the optical quality of eyes by comparison varies markedly (Porter, Guirao, Cox, & Williams, 2001). Our results with mammograms illustrate an important case where the environmental variations are large enough to alter the state of blur adaptation. That is, the “unnatural” visual world that mammograms present through their steepened amplitude spectra is sufficient to recalibrate spatial vision so that the perception of blur and image focus is significantly altered.

Our results also parallel previous findings in showing that adaptation to images with the biased spectra characteristic of natural images alters the CSF by selectively reducing sensitivity to lower spatial frequencies (Bex, Solomon, & Dakin, 2009; Sharpee et al., 2006; Webster & Miyahara, 1997). Again, this is important because the CSF is widely used to predict visibility and visual performance and it is well recognized that it is important to use a measure of the CSF that is appropriate for the viewing conditions of the observer. For example, the CSF varies with factors such as mean luminance, temporal frequency, or location in the visual field (De Valois, Morgan, & Snodderly, 1974; Robson & Graham, 1981; Rovamo, Virsu, & Nasanen, 1978; van Nes, Koenderink, Nas, & Bouman, 1967). The present results confirm that mammograms are not immune to these effects and suggest that reading mammograms may induce a particular state of adaptation.

Our CSF results are also relevant to an existing literature on “eye-filtered” models of detection (Bouwman, van Engen, Dance, Young, & Veldkamp, 2014; Burgess, 1994; Monnin, Bochud, & Verdun, 2010; Monnin, Marshall, Bosmans, Bochud, & Verdun, 2011; Van Peteghem, Bosmans, & Marshall, 2016) These models use a CSF function to modify a simple matched-filter target detector. Most of these use the CSF function suggested by Burgess (1994), which is based on earlier works in vision science (Kelly, 1975; Wilson & Giese, 1977) that did not account for any form of textural adaptation. Our work suggests that these models may be improved by adding more low-frequency suppression in tasks where subjects may be adapting to image backgrounds.

Interestingly, both our measurements here and those in previous studies have found that the effects of adaptation on the CSF are not strongly dependent on the precise slope of the adapting spectra. In particular, as also reported by Webster and Miyahara (1997), the threshold changes were virtually identical whether observers were adapted to a spectral slope of − 1 or − 1.5 and it was only for larger deviations that substantially different CSFs may emerge, at least for the brief timescales assessed in our study. In this regard, the visual world of mammograms is not unnatural – for it appears to induce similar changes in threshold sensitivity.

But how can adaptation to different spectral slopes alter the salience of fine detail in the image (blur after-effects) when it does not seem to alter the sensitivity to fine detail (as measured by the contrast sensitivity function)? The answer to this question is complex but is likely to reflect the complex relationship between threshold sensitivity and suprathreshold appearance. The actual basis of blur perception in the visual system is in fact poorly understood – it is not clear whether blur is coded as a feature that is present in images (e.g. the fuzziness of edges) vs one that is absent (e.g. inability to see expected details). In the former case, it is also unknown whether the attribute of blur is coded as an explicit image feature or implicitly by the pattern of energy across different spatial scales. For example, Elliott et al. (2011) showed that how blur adaptation normalizes subjective judgments of focus can be accounted for by how adaptation alters the balance of responses across multiple narrowly tuned spatial frequency mechanisms.

What our results do support is evidence that subjective judgments of focus and how these are adapted cannot be predicted from the thresholds limiting spatial vision, even though these thresholds do predict blur discrimination (Watson & Ahumada, 2011). There are several arguments for this dissociation (Webster, Mizokami, Svec, & Elliott, 2006). However, among the most telling and that our results demonstrate, is that adapting to an image that is physically in focus induces a large change in the CSF by selectively reducing sensitivity to lower frequencies, but does not cause images themselves to appear sharper.

With regard to target detection, adaptation to the images might have been expected to discount the salience of the background and thus make the targets more conspicuous (McDermott et al., 2010; Wissig et al., 2013). We did not, however, observe this effect and there are again several factors that might be relevant. Our previous results showed that adaptation to the original dense or fatty images did aid detection relative to a pre-adapt baseline, but only when observers were searching on the same images (Kompaniez-Dunigan et al., 2015). The present results suggest that these effects are not selective for the differences in amplitude spectra that we tested. That is, adaptation to both the unfiltered and filtered images might have produced the same effect – as they did on the CSF – so that relative differences between them did not occur. Second, improvements in target salience are only predicted if the target is in fact distinct from the background, so that the background can be selectively suppressed (or the target selectively enhanced). That is, while adaptation may enhance the visibility of novel information, it is not expected to enhance the visibility of stimuli that fall within the adapting distribution (McDermott et al., 2010). Thus, it is possible that adaptation did not facilitate detection of the Gaussian target because the target and background were both affected in similar ways by the adaptation. Again, this is different from the search biases we found when observers were instead adapted to the textural differences between the fatty and dense images, which did reveal search improvements when adapted to the appropriate texture (Kompaniez-Dunigan et al., 2015). The fact that these enhancements did not occur for differences in the amplitude spectra suggest that the search improvements seen with the texture adaptation was not a consequence of simple “learning,” and may instead reflect selectivity of the adaptation to the specific phase spectra of “fatty” and “dense” images relative to the Gaussian targets.

Regardless of the basis for these different after-effects, we have shown that they are manifest in predictable ways in the medical images that radiologists are routinely exposed to. Whether they are also manifest within the context of an actual clinical session or in trained radiologists remains to be tested. However, our work again suggests that adaptation is in principle an important factor in understanding how medical images – or indeed any images – are perceived and could potentially impact decisions based on these perceptions. While there are numerous guidelines for lighting and display specifications in radiological reading rooms (Chawla & Samei, 2007; Harisinghani et al., 2004; Siddiqui, Chia, Knight, & Siegel, 2006), we have noted previously (Kompaniez et al., 2013) that there are few guidelines for specifying how images should be sampled or inspected during a radiological reading. Inspection protocols that control for differential adaptation, such as order effects or exposure history, may aid the visual judgments of the radiologist. Similarly, image processing algorithms that simulate the consequences of adaptation (Webster, 2014), may facilitate medical image perception by pre-adapting and thus optimizing the image for the observer. Finally, how spatial sensitivity is adapted and optimized in the observer may also play a role in understanding the perceptual implications of different imaging modalities, including the differences between projection imaging (e.g. the mammogram images used here) and volumetric imaging such as computed tomography (CT). Radiology has seen a substantial shift towards volumetric imaging over the last 20–30 years, with CT and magnetic resonance imaging replacing projection radiography. Metheany, Abbey, Packard, and Boone (2008) showed that anatomy in breast CT images has an amplitude spectrum that is much closer to a spectral slope of − 1. Chen et al. (2015) compared radiologists’ lesion-detection performance in “thin” slice images compared to “thick” projections of the volumetric data. In addition to overall improved performance for slice images compared to projections, they found that humans had higher efficiency relative to a prewhitened matched filter for the task, suggesting that humans are better at reading the images with a spectral slope close to − 1.

## Conclusions

Exposure to the “blurred” amplitude spectra of mammograms is sufficient to induce adaptation to the blur, altering judgments of image focus and biasing threshold contrast sensitivity. These after-effects parallel the perceptual changes induced by adaptation to textural differences in the images, though the after-effects driven by the amplitude spectrum may be less selective for the differences between images. These changes in sensitivity and perception are important for characterizing and predicting how the visual inspection of medical images is altered by exposure and consequent adaptation to the visual properties of the images.

## Additional file

Additional file 1: An *animation* illustrating blur after-effects in the images. The three adapting images with slopes of − 1.4, − 1.25, or − 1 are shown for 3 s, alternated with three test images all with the same slope of − 1.25 shown for 1 s. The after-effects are best experienced by continuously fixating the *center image*. The test images on the *left* and *right* should appear sharper and blurrier, respectively, relative to the central test image. (GIF 180kb)

## Abbreviations

BIRADS: Breast imaging reporting and data system
CSF: Contrast sensitivity function
