# Supplementary figures and images for: Visual adaptation and the amplitude spectra of radiological images
Source: Cogn Res Princ Implic. 2018 Jan 24;3:3. doi: 10.1186/s41235-018-0089-4 (PMC5783991; doi:10.1186/s41235-018-0089-4)

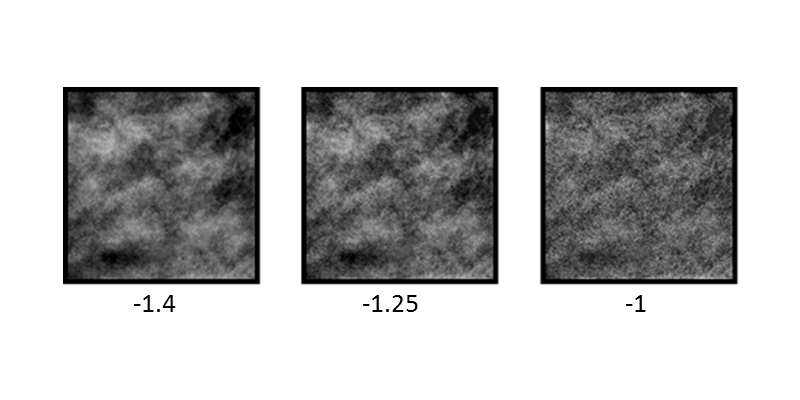

Supplement: Supplementary file 1 — An animation illustrating blur after-effects in the images. The three adapting images with slopes of − 1.4, − 1.25, or − 1 are shown for 3 s, alternated with three test images all with the same slope of − 1.25 shown for 1 s. The after-effects are best experienced by continuously fixating the center image. The test images on the left and right should appear sharper and blurrier, respectively, relative to the central test image. (GIF 180kb) [file 41235_2018_89_MOESM1_ESM.gif]
